# Undergraduate STEM Research: Critical for Competition

**DOI:** 10.1021/acsomega.5c07136

**Published:** 2025-12-30

**Authors:** Jennifer E. Grant

**Affiliations:** 14757 The University of Wisconsin-Stout, Menomonie, Wisconsin 54751, United States

## Abstract

Undergraduate research is a powerful accelerator of STEM leadership, cultivating teamwork, resilience, creativity, and fluency in emerging technologies. By embedding research into undergraduate education, we transform students into innovators and prepare them to push science forward. The real question is not whether undergraduates can do research but whether institutions have the courage to scale it into a modern, competitive practice. How does Artificial Intelligence fit in, and can all undergraduates benefit from having a virtual AI scientific team? The time is now to elevate undergraduate research, nationwide, to a more intentional role in supporting STEM competitiveness.

## Graphical abstract

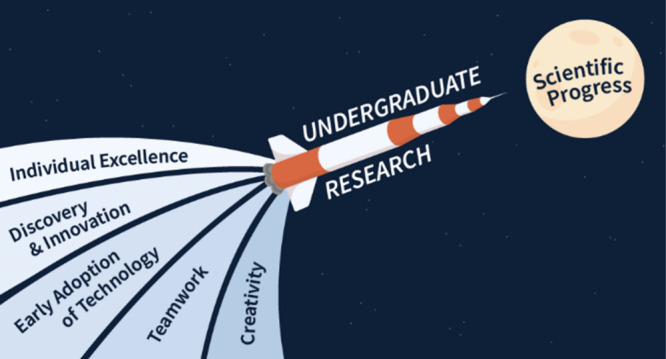

## Introduction

Over two decades of mentoring, I have witnessed how authentic undergraduate research transforms curiosity into resilience and resilience into ingenuity, qualities essential for tomorrow’s scientific leaders. Despite its transformative impact, undergraduate research remains difficult to deploy at a scale that involves most undergraduates. Course-based undergraduate research experiences (CUREs) provide a significant resource, as do traditional research experiences, but a key question for faculty is scalability. Reports from the National Academies emphasize that we need to provide active-learning disciplinary research experience as a critical component in strengthening the STEM workforce.^1^ Similarly, the National Science Board warns that the U.S. risks falling behind in key innovation sectors without robust investments in STEM talent.^2^

In this Viewpoint, I provide insight into innovations in undergraduate research that help future STEM leaders hone grit, teamwork, technological fluency, and creativity.

## Grit and Individual Excellence

We celebrate undergraduate achievement in STEM, but achievement without grit is brittle. Experiments fail, and hypotheses collapse. How do we teach grit? If we value grit, then why do we not have assessments for it?

The Boyer 2030 Commission urges universities to equip undergraduates with skills that transcend disciplinary silos and prepare them for complex societal challenges.^3^ The scientist who will drive the next breakthrough is the one who has learned to navigate uncertainty without losing curiosity. National reports call for adaptability and problem-solving as essential workforce skills. These skills are learned from facing and surmounting challenges. We should measure excellence not just by posters or awards but by persistence in the face of adversity.

Undergraduate research provides the safest arena in which to fail boldly; better here than in graduate school, where stakes are higher. As a faculty member, I find the hardest skill to learn is the wisdom to resist the urge to rescue a student from their own learning. Rescuing a student from an appropriate challenge is premature. And yet, calibrated support is difficult to achieve. We need to spend more time talking about how to encourage students to try something difficult without smothering them.

Imagine an undergraduate system that rewards students for resilience, risk-taking, and adaptability. Such a system would produce not only better scientists but also grittier humans capable of thriving in uncertain times on difficult projects. Grit is not accidental; it is honed by persisting through challenges we think are larger than our abilities. The question is whether we can engineer STEM education to teach this rare skill.

## Teamwork

Where necessity is the mother of invention, intrateam dynamics is the accelerator of ingenuity. National reports emphasize that the STEM workforce must be prepared to solve problems that cross disciplinary boundaries, and teams will have to achieve that collaboratively.

In my lab, students must share ideas in real time, invite critique, and adapt under pressure. Teamwork cultivates accountability, shared ownership, and the ability to innovate under deadlines. These dynamics mirror the realities of professional science, where breakthroughs emerge from diverse teams that leverage complementary expertise. Course-based undergraduate research experiences (CUREs) and digital collaboration platforms now allow students to practice these skills at scale, preparing them not only to participate in STEM but also to compete in it.

## Early Adoption of Technology: The AI Frontier

We are living in a transformative moment where anyone with an Internet connection can wield Artificial Intelligence. From prediction of protein structures to simulation of climate models, AI systems now enable individuals to generate insights without deep expertise in scientific computation. Work that once required weeks or months of computation now may be completed in hours or days. An undergraduate with a laptop can now generate leads that once required a PhD researcher.

This democratization of discovery lowers barriers, accelerates hypothesis generation, and fosters innovation across disciplines.^4^ Initiatives such as the U.S. National AI Research Resource aim to increase access to data sets, models, and computation power nationwide.^5^ We, and our undergraduates need to prepare for this unprecedented level of access to AI computational power. We should applaud this, and we need to embrace it within undergraduate research.

Yet this democratization comes with a paradox. Studies show that while AI enhances efficiency, it can also diminish critical thinking and foster over-reliance.^6^ Without a grounding in theory, students risk becoming prompt engineers rather than scientists. As the National Academies note, AI is emerging as a “scientific partner,” but its use demands careful attention to ethics, equity, and interpretability.^7^

Scientific literacy can no longer mean memorizing formulas alone; it must encompass the ability to critically evaluate AI-generated results and use AI where it has genuine strengths that complement human creativity. And if knowledge itself is not the rate-limiting factor in idea- or intellectual-property generation, then scientific literacy must now require that we teach undergraduates how to lead meaningful problem-solving.

The path forward involves embedding AI into curricula as both a tool and a topic of inquiry. Undergraduates should learn not only how to use AI systems but also when to rely on themselves for leading inquiries and the overall project. Undergraduates, even at the smallest or most modestly funded colleges, benefit from having an unprecedented level of tools and information at their fingertips through their Internet connection. The key for us will be to understand how to help undergraduates thrive using AI as an entire virtual scientific team.

## Creativity

Creativity in science has led to breathtaking innovation and life-saving breakthroughs. Yet a key question remains: how do we teach scientists to be creative?

Creativity emerges when students design their own experimental plan and then adapt and reflect on the outcomes. As the Boyer 2030 Commission report^3^ emphasizes, preparing undergraduates for “world readiness” requires cultivating creativity alongside technical competence, especially in an era where information is instantly accessible.

One undergraduate of mine reframed a decades-old puzzle concerning protein citrullination by integrating statistical modeling with a bioinformatics collaboration that he initiated. This breakthrough exemplifies creativity in action. Creativity flourishes when students combine biomedical questions with agency and collaboration.

The rise of AI further complicates this landscape. If students can instantly retrieve facts, then we should collectively pivot from memorization toward cultivating creativity, critical thinking, and ethical judgment. This aligns with the National Academies’ 2025 report calling for inquiry and innovation.^1^ Creativity serves as the counterbalance to fears that students will develop overreliance on AI; when students are assessed on original problem-solving and design, rote recall becomes de-emphasized.

It is time to deploy a cognitive apprenticeship intentionally, making creativity a core learning outcome of undergraduate STEM education. The challenge is scale. How do we ensure projects are meaningful, and how do we invite an unprecedented percentage of our students to participate? How do we guarantee that students make an investment in creativity? These are the problems we will have to solve.

## Conclusion

To neglect undergraduate research is to concede global leadership; to embrace it is to unleash a generation bold enough to lead discovery.

Undergraduate research, whether in the form of a CURE or a traditional mentored research experience, is mission-critical to STEM leadership. Most of us already support that idea, but I doubt we agree on what this looks like in the 21st century moving toward 2050. We must address the scalability of these experiences and, in effect, democratize access to authentic disciplinary learning experiences. We must come to terms with how AI has already disrupted the STEM education landscape and created a competitive platform the likes of which most of us as faculty have not participated in and likely do not fully comprehend.

If we succeed, we will unleash a generation of scientists resilient in adversity, fluent in emerging technologies, and bold enough to devise solutions to intangible problems.
